# Is Testosterone Replacement Safe in Men with Cardiovascular Disease?

**DOI:** 10.7759/cureus.7324

**Published:** 2020-03-19

**Authors:** Talha Ahmed, May Alattar, Kevin Pantalone, Reyaz Haque

**Affiliations:** 1 Internal Medicine, University of Maryland Medical Center, Baltimore, USA; 2 Endocrinology, University of Maryland, Baltimore, USA; 3 Endocrinology, Cleveland Clinic, Cleveland, USA; 4 Cardiology, University of Maryland, Baltimore, USA

**Keywords:** testosterone replacement therapy, cardiovascular, testosterone hormone, cardiovascular outcomes, current guidelines, hypogonadism

## Abstract

Testosterone is an anabolic hormone that is responsible for the development of male sex organs. It also increases muscle mass and fortifies bone density. In addition to being responsible for primary sexual characteristics at birth and puberty (development and changes of sexual organs such as uterus, vagina, penis, and testes), testosterone is also involved in maintaining secondary sexual characteristics. Patients with low testosterone who are symptomatic should be treated with testosterone replacement therapy (TRT) once the diagnosis has been confirmed. The goal of treatment is to improve the symptoms including the physical, sexual, and cognitive health with the aim being to keep the testosterone in the mid-normal limit of the reference range. Male hypogonadism has been increasingly diagnosed and treated in elderly males since the last decade. A proportionate increase in the prescription of testosterone has been seen as well. The relationship of testosterone levels with cardiovascular (CV) outcomes is challenging and has shown conflicting results. Moreover, in patients with established CV disease, those with high CV risk factors including diabetes, or those with significant risk factors for atherosclerotic CV disease (ASCVD), the benefits of TRT should be weighed against the risks of replacement. Risks and benefits of TRT should be discussed with every patient prior to starting or restarting the procedure.

## Introduction and background

Modern androgen therapy started in 1935 when testosterone was isolated from bull testes and, in the same year, testosterone was chemically synthesized independently. Since then, multiple different forms of prescription have been formulated. Male hypogonadism has been widely diagnosed and treated in elderly males since the last decade [1]. The diagnosis requires two morning testosterone samples to be below 270 ng/dl (reference range: 270-1070 ng/dL). According to one study, 2.4 million men aged between 40 and 69 years suffer from hypogonadism in the United States (US) with an estimated 481,000 new cases of hypogonadism reported annually in men within the same age group. Testosterone levels usually peak at the age of 30 after which there is a decline at a rate of 1-2% per year with aging. Numerous studies have reported decreasing testosterone levels in men with advancing age. Patients with low testosterone who are symptomatic should be treated with testosterone replacement therapy (TRT) once the diagnosis has been confirmed. The goal of treatment is to improve the symptoms including the physical, sexual, and cognitive health with the aim being to keep the testosterone in the mid-normal limit of the reference range. In patients with established cardiovascular (CV) disease, those with high CV risk factors including diabetes, or those with significant risk factors for atherosclerotic CV disease (ASCVD), the benefits of TRT should be weighed against the risks of replacement [2]. The relationship of testosterone levels with CV outcomes is challenging and has shown conflicting results. It is challenging due to the presence of potential confounders like diabetes, obesity, sleep apnea, human immunodeficiency virus (HIV), end-stage renal disease, and chronic obstructive pulmonary disease, which can cause low testosterone (hypogonadism) on one hand and lead to adverse CV events (such as myocardial ischemia, myocardial infarction (MI), stroke, and heart failure) on the other [3]. In light of the current evidence and recommendations, risks and benefits of TRT should be discussed with every patient prior to the initiation of TRT or restarting it in patients who have already suffered a CV event.

## Review

A 61-year-old man with a history of hypertension, hyperlipidemia, obesity, and hypogonadism seeks your advice regarding TRT. He has been on testosterone for a few years. One year ago, his testosterone treatment was stopped after he had presented with an episode of non-ST-elevation MI (NSTEMI). He returns for follow-up after being on guideline-based medical treatment to optimize his blood pressure control and also taking a high-intensity statin and aspirin. He asks you to restart testosterone replacement to help with fatigue and to regain some muscle strength. What should be the best way to proceed?

Overview

Male hypogonadism has been increasingly diagnosed and treated in elderly males since the last decade [4]. According to one study, 2.4 million men aged between 40 and 69 years suffer from hypogonadism in the US with an estimated 481,000 new cases of hypogonadism reported annually in men within the same age group. Testosterone levels usually peak at the age of 30 after which there is a decline at a rate of 1-2% per year with aging. Numerous studies have reported decreasing testosterone levels in men with age. One of them involving both cross-sectional and longitudinal components reported low levels of total testosterone (TT) in up to 20% of men over 60 years, 30% over 70, and 50% over 80 years of age. They suggested that further investigation of testosterone replacement in older men, perhaps targeting those with the lowest serum testosterone concentrations, was justified [5]. The Massachusetts Male Aging Study also showed a decrease in TT with increasing age, particularly when combined with obesity [6].

Physiological effects of testosterone

Testosterone is an anabolic hormone that, in addition to being responsible for primary sexual characteristics at birth and puberty, is involved in maintaining secondary sexual characteristics throughout life. These characteristics include male hair pattern, voice deepening, lean muscle mass, muscle strength, libido, as well as beneficial cognitive effects on mood. It is also involved in erythropoiesis and maintains red blood cell mass. Hence, patients with testosterone deficiency usually experience decreased libido, erectile dysfunction, fatigue, lethargy, decrease in muscle strength, osteoporosis, depression, decreased intellectual function, and regression of some secondary sexual characteristics. Testosterone circulates in the body in a biologically inactive form bound to sex hormone-binding globulin (SHBG) (68%), while its biologically active form exists as loosely bound to albumin (30%) and free testosterone (1-2%) [7]. As people age, the levels of SHBG increase and bind the free testosterone; additionally, rising aromatase enzymes increase the conversion of testosterone to estradiol, both of which decrease the level of the biologically active form of testosterone [8].

Testosterone use in the United States

Modern androgen therapy started in 1935 when testosterone was isolated from bull testes and, in the same year, testosterone was chemically synthesized independently. Since then, multiple different forms of prescription have been formulated. In the 1950s and 1960s, research concentrated on the chemical modification of androgens in order to emphasize their anabolic effects. In the 1970s, the orally effective testosterone undecanoate was added to the spectrum of preparations. However, they were only recently approved by the Food and Drug Administration (FDA) in the US, after first being rejected. Testosterone usually comes in the form of a transdermal patch, gel, foam, ointment, buccal mucosal adhesive, injection, and implantable pellets. With all these new and attractive formulations now available, an impressive increase in testosterone prescription has been noticed, from 0.81% in 2001 to 2.91% in 2010 (more than threefold increase) [9]. Among US men, a trend towards greater testosterone testing, new initiation, and initiation without recent testing has been attributed to televised direct-to-consumer advertising [10].

Who should be treated for low testosterone ‘T’?

Males with symptomatic hypogonadism should be treated with TRT once the diagnosis has been confirmed. The goal of treatment is to improve the symptoms including the physical, sexual, and cognitive health with the aim being to keep the testosterone in the mid-normal limit of the reference range. Patients who have been started on TRT should ideally be followed up in 6-8 weeks to repeat serum testosterone levels and adjust the dose accordingly. Patients getting injections of testosterone usually require earlier monitoring of the testosterone. Any change in dose or route of administration should be followed up in 2-3 months for symptomatic improvement and testosterone level measurement. In addition, prostate-specific antigen (PSA) levels and hematocrit levels should be monitored along with the testosterone levels with goal levels set (depending upon the formulation used). A baseline lipid panel should be done and repeated after 6-12 months [11]. In patients with reversible causes of low testosterone ‘T’, one should aim to modify the underlying cause. This includes patients with morbid obesity who should be counseled regarding weight loss and other modalities to treat obesity, including surgical management if necessary. Patients with sleep apnea tend to have a reversible cause of hypogonadism and compliance with continuous positive airway pressure (CPAP) should be encouraged in them. This will not only improve their quality of life but may also mitigate the need for TRT initiation.

Testosterone use in patients with cardiovascular risk factors

In patients with established CV disease or those with increased CV risk factors including risks for ASCVD, the benefits of TRT should be weighed against the risks of replacement. Over the past few years, the FDA has concluded that there is no evidence for significant CV risk for any given group of people treated with TRT. However, further studies are warranted to elucidate the risks of TRT in patients with significant CV risk factors and those with prior history of CV events. Recommendations from both the American College of Cardiology (ACC) and American Endocrine Society are to avoid TRT in men with poorly controlled heart failure (HF), recent myocardial infarction (MI), revascularization, and stroke within last six months [12].

Clinical evidence

The relationship of testosterone levels with CV outcomes is challenging and has shown conflicting results. It is challenging due to the presence of potential confounders like diabetes, obesity, sleep apnea, human immunodeficiency virus (HIV), end-stage renal disease, and chronic obstructive pulmonary disease, which can cause low testosterone on one hand and lead to adverse CV events on the other. Results from animal studies have shown varying mechanisms of action for testosterone, making things even more complex. It has cardio-protective, vasodilatory, and anti-inflammatory properties but, at the same time, has been shown to have vasoconstrictive, pro-atherosclerotic, and pro-inflammatory properties [13].

The current ACC/American Heart Association (AHA) recommendations are not very clear about the relationship between TRT and CV events including angina, MI, stroke/transient ischemic attack (TIA), and sudden cardiac death [14]. From observational studies and meta-analyses, it is evident that patients with CV events have low testosterone, which might be an indicator of poor health in general. In 2015, however, based on the results of studies highlighting the adverse CV effects of testosterone replacement, the FDA Drug Safety Communication cautioned that prescription testosterone products should only be approved for men with low testosterone caused by medical disease. They also concluded that there is a possibility of increased CV risk associated with testosterone use. The benefits of treating low testosterone ‘T’ due to aging are still not proven and controversies persist.

Current recommendations

The current guidelines recommend no clear evidence of CV risk for any given group of patients treated with TRT. However, its safety in patients with CV risks and previous CV events is still doubtful due to recent studies showing conflicting results for TRT in patients with adverse CV risk factors. Hence, the ACC guidelines now recommend avoiding TRT in patients with recent MI, revascularization, advanced or severe heart failure, and stroke within the last six months [15]. However, there still is a need for prospective randomized control trials where patients are followed for at least a year or more, including patients with a valid diagnosis of hypogonadism, using two standardized early-morning testosterone samples.

## Conclusions

Risks and benefits of TRT should be discussed with every patient prior to starting the procedure. Our patient, in particular, had a CV event in the past year, and the main focus was on the aggressive management of modifiable atherosclerotic risk factors. Our approach was to screen the patient again at interval clinic visits to confirm the clinical and biochemical evidence of hypogonadism. As his last CV event had occurred six months ago, in light of the current evidence and ACC/AHA recommendations, and also considering our patient's persistent symptoms, he was restarted on TRT with routine follow-up.

## References

[1] Laurent MR, Antonio L, Vanderschueren D (2016). Testosterone treatment in older men. N Engl J Med.

[2] Gencer B, Mach F (2016). Testosterone: a hormone preventing cardiovascular disease or a therapy increasing cardiovascular events?. Eur Heart J.

[3] Corona G, Maseroli E, Rastrelli G, Isidori AM, Sforza A, Mannucci E, Maggi M (2014). Cardiovascular risk associated with testosterone-boosting medications: a systematic review and meta-analysis. Expert Opin Drug Saf.

[4] Baillargeon J, Urban RJ, Kuo YF (2014). Risk of myocardial infarction in older men receiving testosterone therapy. Ann Pharmacother.

[5] Harman SM, Metter EJ, Tobin JD, Pearson J, Blackman MR (2001). Longitudinal effects of aging on serum total and free testosterone levels in healthy men. J Clin Endocrinol Metab.

[6] Araujo AB, O'Donnell AB, Brambilla DJ, Simpson WB, Longcope C, Matsumoto AM, McKinlay JB (2004). Prevalence and incidence of androgen deficiency in middle-aged and older men: estimates from the Massachusetts Male Aging Study. J Clin Endocrinol Metab.

[7] Lu W, Guo W, Cui D, Dong K, Qiu J (2019). Effect of sex hormones on brain connectivity related to sexual function in perimenopausal women: a resting-state fMRI functional connectivity study. J Sex Med.

[8] Matsumoto AM (2020). Testosterone replacement in men with age-related low testosterone: what did we learn from the Testosterone Trials?. Curr Opin Endocr Metab Res.

[9] Basaria S, Coviello AD, Travison TG (2010). Adverse events associated with testosterone administration. N Engl J Med.

[10] Vigen R, O’Donnell CI, Barón AE (2013). Association of testosterone therapy with mortality, myocardial infarction, and stroke in men with low testosterone levels. JAMA.

[11] Sharma R, Oni OA, Gupta K (2015). Normalization of testosterone level is associated with reduced incidence of myocardial infarction and mortality in men. Eur Heart J.

[12] Diem SJ, Greer NL, MacDonald R (2020). Efficacy and safety of testosterone treatment in men: an evidence report for a clinical practice guideline by the American College of Physicians. Ann Intern Med.

[13] Budoff MJ, Ellenberg SS, Lewis CE (2017). Testosterone treatment and coronary artery plaque volume in older men with low testosterone. JAMA.

[14] Kloner RA, Carson C 3rd, Dobs A, Kopecky S, Mohler ER 3rd (2016). Testosterone and cardiovascular disease. J Am Coll Cardiol.

[15] Corona G, Torres LO, Maggi M (2020). Testosterone therapy: what we have learned from trials. J Sex Med.

